# Unusual Site of a Bizarre Parosteal Osteochondromatous Proliferation (Nora’s Lesion) Involving the Scapula: First Case Report and Review of the Literature

**DOI:** 10.7759/cureus.38980

**Published:** 2023-05-13

**Authors:** Ramy Samargandi

**Affiliations:** 1 Orthopedic Surgery Department, College of Medicine, University of Jeddah, Jeddah, SAU

**Keywords:** benign tumors, periosteal enchondroma, periosteal chondrosarcoma, nora's lesion, bizarre parosteal proliferation of bone

## Abstract

Bizarre parosteal osteochondromatous proliferation (BPOP), also known as Nora's lesion, is a rare benign surface lesion of bone that typically occurs in the hands and feet. We report herein the first case of BPOP occurring in an unusual location, specifically the scapula of a 29-year-old male patient. The lesion exhibited features mimicking those of a peripheral chondrosarcoma because of its atypical location in the axial skeleton and the presence of calcification, which indicates the presence of cartilaginous matrix. Treatment involved a wide surgical resection, and histopathological examination confirmed the diagnosis of BPOP of the bone. At a five-year follow-up, there was no evidence of local recurrence.

## Introduction

Bizarre parosteal osteochondromatous proliferation (BPOP), also known as Nora's lesion, is a rare, benign osseous lesion characterized by a parosteal mass containing cartilage, fibrous, and bone tissues [1,2]. It was first described by Nora et al. in 1983, who reported 35 cases affecting mainly the hands and feet [3]. Although it can occur at any age, BPOP most commonly presents in the third and fourth decades, with no gender predilection [1-3]. While malignant transformation and metastasis have not been reported, there is a high risk of recurrence following surgical resection, ranging between 20% and 55% [1,4]. BPOP typically presents with pain and bony swelling that progressively increases in size and typically occurs without a history of trauma [1,4,5]. Accurate diagnosis is essential to guide treatment strategy, as BPOP can resemble other benign and malignant lesions such as osteochondroma, myositis ossificans, periosteal chondrosarcoma, and parosteal osteosarcoma [5,6]. To date, over 200 cases of BPOP have been documented in the literature, with the hands and feet being the most common sites, followed by the long bones [1,3]. Other rare sites have also been reported, such as the skull, maxilla, mandible, sesamoid bone, and vertebrae [1,5,7-9]. In this report, we present the first case of BPOP involving the scapula, specifically the scapular spine.

## Case presentation

History and clinical presentation

A 29-year-old right-handed male with an unremarkable medical history presented with a six-month history of a posterior left scapular mass. Initially non-painful, the mass had increased in size over the past two months and was now accompanied by mild pain that was exacerbated by lying down and palpation. There was no history of trauma or infection. Physical examination revealed a palpable, tender, firm, and immobile mass in the middle portion of the scapular spine with normal overlying skin. No other palpable mass was found at other sites. Range of motion was not limited, and there were no deficits on the neurovascular examination. Other general physical and systemic examinations were normal.

Investigation

**Figure 1:**
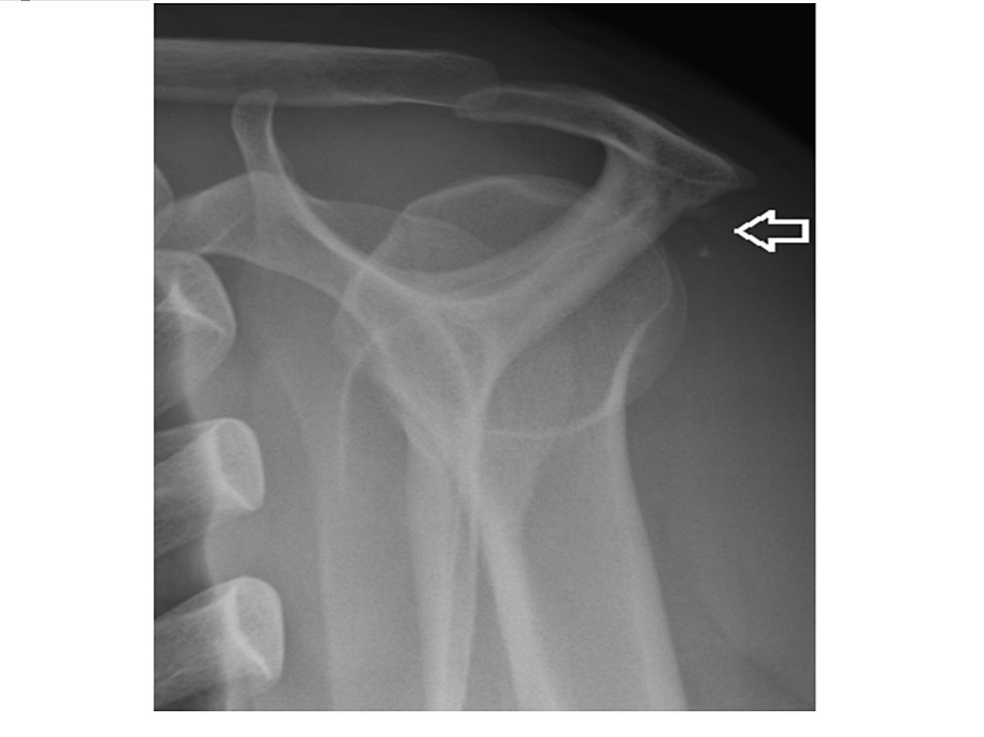
Lateral Y view radiograph of the shoulder shows a juxta-cortical calcific lesion in the spine of the scapula (arrow)

**Figure 2:**
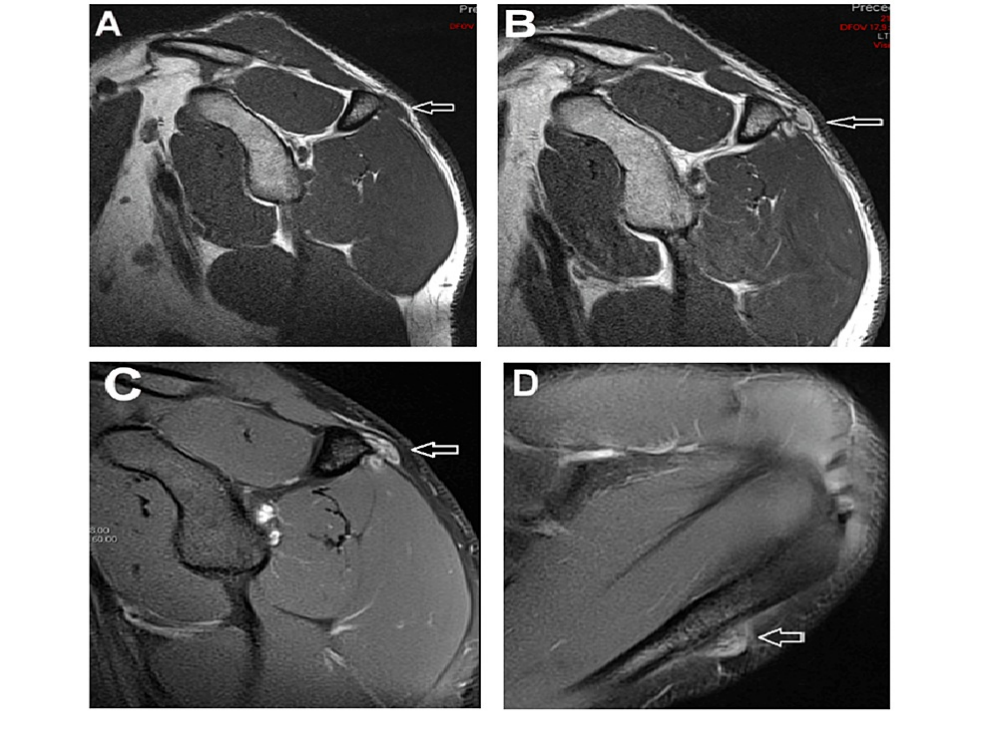
MRI findings of the lesion (white arrow): (A) Sagittal T1W sequences demonstrate an isointense lesion similar to muscles. (B) Sagittal T1W sequence after gadolinium injection shows heterogeneous uptake, with some areas of low signal intensity indicating calcification. (C) Sagittal fat-suppressed T2W image shows a heterogenous lesion with a high signal area and areas of low signal, with an intact cortical bone. (D) Axial fat-suppressed T2W image illustrates the juxtacortical position of the lesion with no direct continuity with the underlying marrow of the bone.

The routine laboratory work-up was unremarkable. Radiographs, including a scapular Y view, demonstrated an ossified lesion adjacent to the scapular spine without evidence of bony erosion or osteolysis of the scapula (Figure 1). Magnetic resonance imaging (MRI) revealed a nodular lesion measuring 16x16x10 mm attached to the cortex of the scapular spine. The lesion appeared iso-intense on T1-weighted images and heterogeneously moderately hyperintense on fat-suppressed T2-weighted images, with some areas of low intensity. There was moderate uptake on gadolinium with no evidence of marrow edema or medullary bone continuity, and no evidence of a cartilage cap was seen (Figure 2). Differential diagnoses included osteochondroma, myositis ossificans, periosteal enchondroma, turret exostosis, and florid reactive periostitis, but other malignant tumors such as periosteal chondrosarcoma and parosteal osteosarcoma could not be excluded.

Surgical management

After a discussion on the multidisciplinary tumor board, the decision was made to perform an excisional biopsy with wide margins without the need for a prior biopsy to avoid biopsy tract contamination. Surgical excision was done under general anesthesia. The lesion was accessed through a direct posterior approach over the mass. Careful consideration was given to achieve a surgical resection with a layer of the scapular spine cortex and safe soft tissue margins. The resection specimen was sent for histopathological examination.

Histopathology

**Figure 3:**
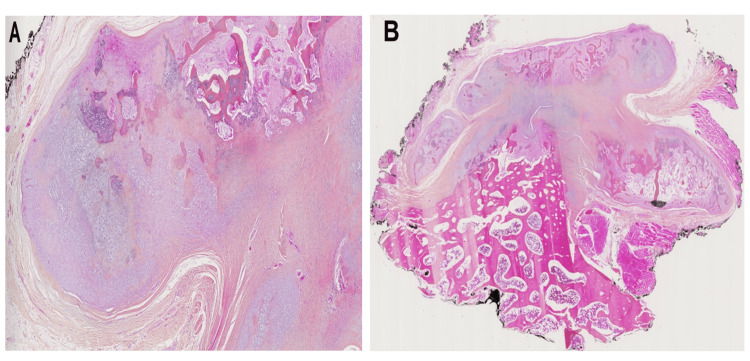
Histopathological findings: (A) Excision specimen of the lesion: on microscopic examination at low magnification, the lesion develops on the surface of the cortex. (B) At higher magnification, the "cap" of the lesion is made up of the disordered interweaving of cartilaginous, fibrous, and blue bone components. Enchondral ossification is focally observed.

Histologically, the lesion exhibited three distinct components, namely fibrous tissue, cartilage, and bone. The fibrous component showed no evidence of atypia, while the cartilage component exhibited enlarged and bizarre chondrocytes with heavy purplish blue mineralization, which is characteristic of the so-called blue bone (Figure 3). In order to rule out the possibility of parosteal osteosarcoma, we performed fluorescence in situ hybridization (FISH) to examine for *MDM2 *gene amplification, which turned out to be negative. The final diagnosis was confirmed to be a bizarre parosteal osteochondromatous proliferation (Nora's lesion), and complete negative margins were achieved.

Post-operative course, outcome, and follow-up

The patient was placed in a simple sling for one week to alleviate pain. However, the patient was found to have a non-displaced scapular body fracture as a result of an inadvertent movement during physiotherapy. The patient was managed conservatively with immobilization and regular follow-up visits, resulting in a full recovery. The patient's shoulder range of motion returned to normal. The patient underwent annual MRI follow-up for five years, and no evidence of recurrence was detected during this period.

## Discussion

BPOP is a rare benign surface lesion most commonly present in the hands and feet, followed by the long bone. In the literature, unusual localizations have also been reported [5,7-9]. To our knowledge, this is the first reported case involving the scapula. A BPOP lesion may be confused with benign and malignant tumors, especially when it presents at unusual sites, as in our case. Common differential diagnoses of BPOP to be considered are malignant lesions such as parosteal osteosarcoma or chondrosarcoma and benign lesions such as myositis ossificans, periosteal chondroma, and osteochondroma. In addition, other benign reactive lesions like turret exostosis and florid reactive periostitis may be added to the differential diagnosis [4-6,10], although some authors believe that these lesions, along with BPOP, are all part of the same pathological spectrum [11,12]. In our case, the lesion mainly mimicked peripheral chondrosarcoma due to its location, as cartilaginous lesion has more malignant potential in the axial skeleton than in appendicular locations. After discussion in the tumor board, it was decided to proceed with surgical resection without prior biopsy to avoid biopsy tract contamination and as the lesion was easily accessible for resection with obtaining negative margins. As the lesion was small, a CT-guided biopsy would have been recommended otherwise [13].

Radiologically, BPOP is characterized by a well marginated mineralized lesion situated on the surface of bone without cortical erosion [1-3,6]. CT scan or MRI is essential for characterizing the lesion and delineating if there is continuity with the medullary cavity, which is an important radiological feature to differentiate between BPOP from osteochondroma. In pedunculated osteochondroma, there is continuity with the intramedullary canal, while a lack of intramedullary continuity is seen on BPOP in most cases [1,3,6]. Although, in rare cases, BPOP may present with intramedullary continuity; it's not a critical, distinctive radiological feature [5]. Histologically, BPOP contains three components: cartilage, bone, and fibrous tissue. The cartilage component is hypercellular with enlarged bizarre chondrocytes but without cytological atypia. The fibrous component has no cellular atypia. The bone component has the characteristic of a purplish-blue mineralization, so-called "blue bone," but with no cellular atypia [1-3]. Although BPOP has not been previously reported in the scapular bone, the histological features of our case were consistent with those described in previously published studies. Moreover, it's important to eliminate parosteal osteosarcoma; histologically, the absence of cellular atypia and the absence of *MDM2* gene amplification by FISH can help to rule out parosteal osteosarcoma, as in our case [14].

The etiology of BPOP is still unknown. It remains controversial whether BPOP is a reactive proliferative process or a neoplastic lesion. Although most BPOP present with no history of trauma, some authors suggest that it may be due to a repetitive trauma [11,12]. However, some studies have shown that BPOP is associated with cytogenetic abnormalities with chromosomal translocations involving t(1;17)(q32;q21), t(1;17)(q42;q23), and inversion of chromosome 7. Therefore, some authors suggested that BPOP is a neoplastic lesion rather than a reactive proliferative process, and cytogenetic studies may help with the correct diagnosis in difficult cases [15-17].

The mainstay of treatment for BPOP is surgical resection [4]. Although there are no reported cases of malignant transformation or metastasis, follow-up is necessary due to the high risk of recurrence, which has been reported as 29% to 55% within the first two years following the initial treatment [1,3,6]. In our case, there was no recurrence observed during the five-year follow-up period, and follow-up was subsequently discontinued. However, we advised the patient to contact our department if there is any suspicion of local recurrence identified through self-palpation.

## Conclusions

BPOP is a rare osseocartilaginous lesion that is usually managed through surgical intervention. Although it is more frequently seen in the hands and feet, there have been reported cases of BPOP in other locations. To our knowledge, this is the first case of BPOP reported in the scapula. Therefore, BPOP should be included in the list of differential diagnoses for surface bone lesions of the scapula.
